# Mutually beneficial host exploitation and ultra-biased sex ratios in quasisocial parasitoids

**DOI:** 10.1038/ncomms5942

**Published:** 2014-09-12

**Authors:** Xiuyun Tang, Ling Meng, Apostolos Kapranas, Fuyuan Xu, Ian C.W. Hardy, Baoping Li

**Affiliations:** 1 School of Plant Protection, Nanjing Agricultural University, No. 1 Weigang, Nanjing, Jiangsu 210095, China; 2 School of Biosciences, University of Nottingham, Sutton Bonington Campus, Loughborough LE12 5RD, UK; 3 Department of Biology, National University of Ireland Maynooth, County Kildare, Ireland; 4 Forest Academy of Jiangsu Province, Dongshanqiao, Nanjing, Jiangsu 211153, China

## Abstract

Selfish interests usually preclude resource sharing, but under some conditions collective actions enhance *per capita* gains. Such Allee effects underlay early explanations of social evolution but current understanding focusses on kin selection (inclusive fitness). We find an Allee effect that explains unusual quasisociality (cooperative brood care) among parasitoid wasps without invoking or precluding kin selection effects. In *Sclerodermus harmandi*, individual females produce most offspring when exploiting small hosts alone. However, larger hosts are more successfully exploited by larger groups of females, with the per-female benefits outweighing the costs of host sharing. Further, the extremely biased sex ratios (97% female) are better explained by mutually beneficial female–female interactions that increase the reproductive value of daughters (local resource enhancement), rather than by the usually invoked local mate competition between males. Thus, atypical quasisocial behaviour in a parasitoid wasp directly enhances reproductive success and selects for very extremely female-biased sex ratios.

## Abstract (web summary)

The evolution of social behaviour is usually explained by kin selection. Here, the authors show that mutual host exploitation by a parasitoid wasp maximizes the average reproductive success of individual females, which suggests that cooperative brood care does not rely on kin selection in these wasps.

## Graphical abstract

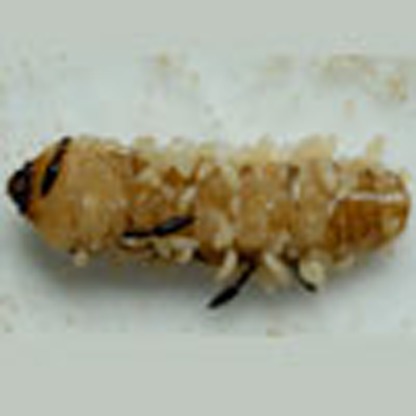

The selfish interests of individuals usually preclude them from opting to share resources, but under some conditions collective actions enhance *per capita* gains: such Allee effects on reproduction can explain mutually beneficial interactions and formed the basis of early explanations of social behaviour^1^,^2^. Most current studies of social evolution utilize kin selection theory based on genetic relatedness (inclusive fitness)^3^,^4^,^5^. Eusocial insects, with cooperative brood care, reproductive division of labour and overlapping generations provide the major empirical focus for these studies^4^,^6^,^7^ and also key tests of inclusive fitness-based sex allocation theory^6^,^8^,^9^, but there are other social insects that lack some eusocial life-history characteristics^2^,^10^.

Some parasitoid wasps exhibit aggressive brood defence by guarding mothers^11^, classing them as sub-social^10^, but few exhibit more complex social biologies and most, lacking parental care, are socially solitary^10^,^12^. In contrast, the quasisocial bethylid wasp *Sclerodermus harmandi* and some of its congeners are among the most socially complex parasitoids known^13^,^14^,^15^: not only do females typically exhibit maternal care^16^,^17^, but multiple foundress females may tend a group of offspring developing on a single host^13^,^18^,^19^ (Fig. 1) and mothers do not exclusively care for their own offspring^16^.

Adult female *S. harmandi* are highly active in searching for host larvae in the field^20^,^21^,^22^,^23^. Once a host is found, females first inspect it with their antennae and mandibles, paralyse it by stinging with the ovipositor, clean the host’s body surface, then feed on the host haemolymph before laying eggs if the host is sufficiently large^23^,^24^,^25^,^26^,^27^. Although larger hosts are preferred for oviposition, they require more time to paralyse, and parasitism rates and offspring survival are both lower than when eggs are laid on smaller hosts^28^. Field evidence shows that multiple females may attack a single long-horned beetle host (with, for instance, up to four females observed attacking single *Saperda populnea* larvae (*ca.*15 mm in body length)^29^. Under multifemale attack, less time is required for paralysis and parasitism rates can be higher than under single-female attack^30^,^31^. After laying eggs, females tend the brood until the progeny mature^16^,^17^,^25^,^32^: as with host attack, brood tending can be performed by single or multiple females^16^,^17^,^25^ with broods on large hosts tended by multiple females producing more progeny than those tended by single females^31^.

Here we provide experimental evidence that mutual host exploitation by *S. harmandi* maximizes the average reproductive success (direct fitness) of individual females, thus explaining quasisociality in this species without recourse to kin selection. We also show that the very extremely female-biased sex ratios observed are better explained by local resource enhancement (LRE)^9^,^33^,^34^, due to mutually beneficial foundress–foundress interactions contributing to the value of female offspring, than by the much more widely applied explanation of local mate competition (LMC) between males^9^,^33^,^35^,^36^.

## Results

### Oviposition and development

The percentage of hosts that were oviposited on was 66.3% overall. The probability of oviposition decreased as host size increased (logistic analysis of covariance (ANCOVA): *G*_1_=14.43, *P*<0.001, %deviance explained=5.1, *n*=220) and as foundress number increased (logistic ANCOVA: *G*_4_=19.18, *P*<0.001, %dev=27.3, *n*=220); there was no significant interaction between these effects (logistic ANCOVA: *G*_4_=0.28, *P*=8.93, *n*=220). The time taken for females to begin ovipositing on a host ranged from 3 to 20 days, with longer periods for larger hosts (ANCOVA: *F*_1,140_=6.14, *P*=0.014, %dev=2.1, *n*=146) and with shorter periods with larger foundress numbers (ANCOVA: *F*_4,140_=36.53, *P*<0.001, %dev=50.3, *n*=146) but with no interaction between host weight and foundress number (ANCOVA: *F*_4,136_=0.94, *P*=0.446, *n*=146). The clutch sizes laid by single foundresses ranged from 6 to 86 eggs (*n*=20). Overall, up to 271 eggs were laid on a host with the number of eggs increasing with both host body weight (log-linear ANCOVA: *F*_1,140_=15.6, *P*<0.001, %dev=4.35, *n*=146) and foundress number (log-linear ANCOVA: *F*_4,141_=50.64, *P*<0.001, %dev=56.54, *n*=146), without significant interaction (log-linear ANCOVA: *F*_4,136_=0.45, *P*=0.773, *n*=146) (Fig. 2). Exploring the clutch size data in terms of the mean number of eggs laid per foundress showed that this average increased with host weight (log-linear ANCOVA: *F*_1,140_=9.96, *P*=0.002, *n*=146) and decreased with foundress number (log-linear ANCOVA: *F*_4,140_=24.12, *P*<0.001, *n*=146), without significant interaction (log-linear ANCOVA: *F*_4,136_=0.19, *P*=0.941, *n*=146). So, provided that eggs were laid, more eggs were laid on larger hosts and by larger groups of females, but the average contribution of eggs by individual females was smaller when foundress groups were larger.

Total developmental time from egg to adult emergence ranged between 23 and 31 days and was shorter on smaller hosts (ANCOVA: *F*_1,109_=8.11, *P*<0.005, *r*^2^=0.05, *n*=112) and shorter in broods developing from larger numbers of eggs (ANCOVA: *F*_1,109_=32.48, *P*<0.001, *r*^2^=0.22, *n*=112); the interaction between these main effects was marginally nonsignificant (ANCOVA: *F*_1,108_=3.62, *P*=0.060, *n*=112). The developmental time was also shorter when offspring were produced by more foundresses (analysed separately to avoid multicollinearity with numbers of eggs laid, ANCOVA: *F*_4,107_=6.04, *P*<0.001, *r*^2^=0.18, *n*=112). Mortality of offspring during development was common, with 67.17% (s.e.m.+2.13, −2.21) of eggs dying before adulthood. Mortality was highly overdispersed (heterogeneity factor=37.0) indicating a tendency for offspring on a given host to survive or die collectively. Developmental mortality was unrelated to host weight (logistic ANCOVA: *F*_1,139_=0.78, *P*=0.378, %dev=0.5, *n*=146), number of eggs laid (logistic ANCOVA: *F*_1,140_=1.98, *P*=0.162, %dev=1.3, *n*=146) or foundress number (logistic ANCOVA: *F*_4,141_=1.96, *P*=0.104, %dev=5.2, *n*=146). The size (mean individual weight) of adult female offspring increased with increasing host weight (ANCOVA: *F*_1,109_=6.88, *P*=0.010, *r*^2^=0.05, *n*=111) but was not significantly affected by the numbers of adults produced per host (an index of resource availability, ANCOVA: *F*_1,108_=2.52, *P*=0.116, *r*^2^=0.02, *n*=111) or by the interaction between host weight and adult offspring number (ANCOVA: *F*_1,107_=3.07, *P*=0.083, *n*=111).

### Offspring production

The probability of producing adult offspring from a given host was enhanced when foundress groups were larger (logistic ANCOVA: *G*_4_=17.06, *P*<0.001, %dev=22.3, *n*=220, Fig. 3a). Enhanced offspring production was due to higher probability of oviposition (logistic ANCOVA: *G*_4_=19.18, *P*<0.001, %dev=27.3, *n*=220) and greater number of eggs being laid (log-linear ANCOVA: *F*_4,140_=50.64, *P*<0.001, %dev=56.5, *n*=220) when foundress groups were larger and not due to developmental mortality of offspring, which was unrelated to foundress number (logistic ANCOVA on grouped binary data: *F*_4,141_=1.96, *P*=0.104, %dev=5.2, *n*=220).

Fitness gains per foundress were contingent on an interaction between foundress number and host weight (log-linear ANCOVA: *G*_4_=4.80, *P*<0.001, %dev=7.8; main effects: host weight, *G*_1_=3.23, *P*=0.074, %dev=1.3; foundress number, *G*_4_=3.00, *P*=0.019, %dev=4.9, *n*=220). Individual foundresses were most productive when attacking small hosts (≤ *ca.* 0.2 g) alone. For intermediate-sized hosts (*ca.* 0.2–0.33 g), foundresses benefited most by being in groups of two or four foundresses, whereas for the largest hosts (>0.33 g), females benefited most by being in groups of eight foundresses (Fig. 3b). Differences in per foundress offspring production within each host size category (Fig. 4), defined according to where the uppermost regression lines on Fig. 3b cross, were explored using three separate Kruskal–Wallis tests. For small hosts, differences in mean ranking were not significant due to limited sample size (*n*=17; *H*=1.229) and for medium hosts production did not differ between foundress number categories (*n*=105, *H*=2.676, *P*=0.613). For large hosts, production differed significantly (*n*=98, *H*=31.47, *P*<0.001) and *post hoc* multiple comparison testing (with critical values calculated for the 10 possible comparisons using: *α*=0.05, *k*=5 groups, *z*=2.807) showed that this difference was due to groups of eight foundresses being significantly more productive than single foundresses (difference in mean ranking=41.11, *P*<0.05) and two-foundress groups (difference in mean ranking=31.09, *P*<0.05), and also to groups of four foundresses being more productive than single foundresses (difference in mean ranking=25.95, *P*<0.05).

### Sex ratios

The sex ratios of groups of maturing adults were very strongly female biased (mean proportion males=0.032,±s.e.m.=0.004, *n*=110 offspring groups). Maternal sex ratio optima are predicted, and observed in many species, to respond to foundress number when offspring develop in discrete groups^9^,^12^,^35^. In *S. harmandi* offspring group sex ratios increased as the number of foundresses increased (logistic regression corrected for overdispersion, heterogeneity factor=1.30: *F*_1,108_=15.79, *P*<0.001, %dev=12.75, *n*=110, Fig. 5) but for multiple foundress cases sex ratios were far more biased than would be predicted by classical LMC theory. Figure 5 contrasts observed sex ratios with predicted evolutionarily stable sex ratio responses to foundress number in haplodiploid species with single-generation mating groups^9^,^35^. While these models assume foundresses are unrelated, some extended LMC models predict increased female bias when foundresses are related, but the differences from classical predictions are not large^9^,^37^.

Mechanistically, the relationship between sex ratio and foundress number appears to be due to individual foundresses producing a constant number of males (mean=0.797, s.e.m.=+0.073, −0.067) irrespective of foundress number (log-linear regression: *F*_1,108_=3.04, *P*=0.084, %dev=2.7), but fewer females when in larger foundress groups (log-linear regression: *F*_1,108_=36.82, *P*<0.001, %dev=26.5, *n*=110), tallying with Mamaev’s^15^ observation that 1–2 males are included among the first eggs laid in *Sclerodermus* clutches.

## Discussion

Multiple foundress oviposition is generally disadvantageous to individual parasitoid mothers, due to subsequent resource competition among developing offspring, and is usually adaptive only when single-foundress opportunities are rare^12^,^38^,^39^,^40^. Where present, Allee effects are usually reported in relation to single (foundress) females laying multiple eggs to suppress host immune defences^41^ or in terms of large offspring groups being required to consume the host and complete development^42^. In contrast, in *S. harmandi* an Allee effect operates via a foundress number-dependent probability of parasitizing (ovipositing on and obtaining offspring from) larger hosts. Our data thus constitute an experimental demonstration of mutually beneficial host exploitation among adult parasitoid wasps, and an independent evolution of cooperative reproductive behaviour within the Hymenoptera likely promoted by phylogenetic constraints on body size and clutch size^43^. Our results also accord with prior reports that larger hosts, once suppressed, enhance reproductive success for single *Sclerodermus* females but also take longer to handle and are more likely to kill the attacking parasitoid than are smaller hosts^13^,^26^,^28^,^30^,^31^,^44^.

*S. harmandi* sex ratios were very strongly female biased, as reported in other studies of this species and its congeners^13^,^15^,^16^,^18^,^28^,^32^,^44^,^45^. Female-biased sex ratios are often explained by the reproductive value of nondispersing male offspring being reduced (by male–male competition for mates, LMC), compared with dispersing daughters^9^,^35^,^46^. LMC models can be formulated in several equivalent ways, with an inclusive fitness approach considered the most flexible^9^,^12^,^33^. Further, under LMC, sex ratios are predicted, and often observed, to become less biased as foundress numbers increase due to sons being progressively able to mate with the daughters of other foundresses^9^,^12^,^35^. Although the observed increase in *S. harmandi* sex ratio with increasing foundress number is in qualitative agreement with expectations from classical LMC theory, and also with anecdotally reported trends for *Sclerodermus pupariae*^44^, the quantitatively small effect of foundress number, coupled with the greater extent of the sex ratio bias than would be predicted (Fig. 5), casts doubt on whether LMC can fully explain these results. LMC could best explain the observed sex ratios of *S. harmandi* if only one dominant foundress produced offspring on each host irrespective of the number of other females present, which could be determined using molecular genetic markers^36^. However, the facts that the total numbers of eggs laid on a host were greater when more foundress were present (Fig. 2), and that females that have not laid their own eggs do not tend broods^16^,^17^ strongly suggest that such extreme reproductive skew is unlikely.

**Figure 1:**
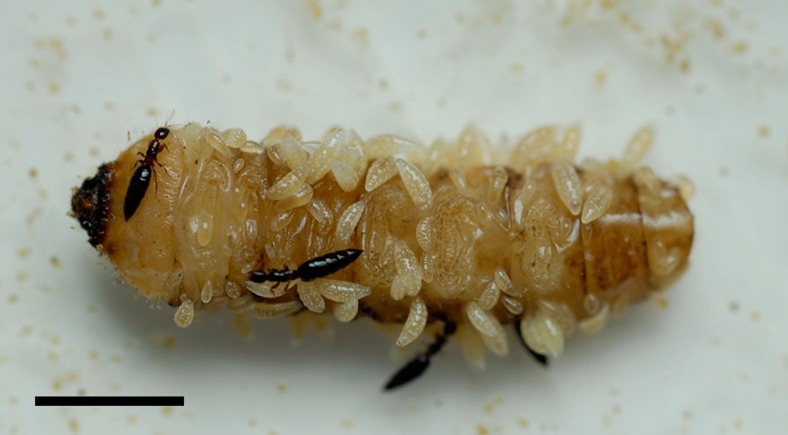
Multiple cofoundresses engaged in cooperative brood care. Adult *Sclerodermus harmandi* females tending a brood of larvae developing on a pine sawyer beetle (*Monochamus alternatus*) larva. Scale bar, 5 mm.

Male–male combat interactions can intensify LMC and lead to greater female bias in some parasitoids^47^,^48^, but combat is not observed in *S. harmandi* or its congeners. Furthermore, *S. harmandi* females are typically wingless (Fig. 1) and males winged^32^, which is expected to promote more intrasex interactions between female than male siblings, due to a greater dispersal of males^9^,^33^,^35^. If females competed for hosts, local resource competition (the generalized form of LMC)^9^,^33^ would be expected to select for decreased investment in females^49^, but given that we have shown that the reproductive value of females is enhanced by mutualistic exploitation of large hosts, LRE, when the production of one sex increases the fitness of relatives more than does production of the other^9^,^33^,^34^,^50^, offers explanation for the extreme female bias. Our results thus indicate that female-biased sex ratios are favoured because they increase the probability that a daughter’s future environment will contain more females with which she can more successfully exploit hosts. The extent of any selected bias is likely to be influenced by factors such as the mating capacity of males and the number and relatedness of the foundress females.

LRE is more often documented in vertebrates and is typically based on intergenerational interactions, such as offspring helping at the parental nest^9^. Among invertebrates, LRE is a strong candidate explanation for female bias in some solitary and facultatively quasisocial bees, with the fitness enhancement operating via multiple-foundresses reducing the probability of post-oviposition parasitism or predation of offspring^21^,^51^. We know of no prior reports of LRE operating in parasitoid wasps. While our results indicate LRE, they contrast with offspring production patterns under LRE in allodapine bees in which production per female follows a dome-shaped relationship with the number of cooperating females^34^. In *S. harmandi* offspring production per foundress declines with foundress number (log-linear regression: *F*_1,109_=35.91, *P*<0.001, %dev=24.8, *n*=111) for cases in which at least some offspring survived, for comparison with analysis in Schwarz^34^. Mechanistically, this difference may be due to the number of failed nests in allodapine bees being unknown^34^, while our data on offspring production comprised both successful and unsuccessful attempts at host suppression. Biologically, the differences may be due to the allodapine bee mutualisms deriving from reduced predation of offspring^34^,^51^,^52^ and being relatively weakly coupled to offspring food supply, while in *S. harmandi* the mutualism is facultatively based on the acquisition of a subsequently limited and shared food resource.

After parasitizing a host, foundress *S. harmandi* females remain in close association with each other and each other’s offspring for extended periods, tending and moving eggs and larvae^13^,^14^,^16^,^17^,^25^,^31^. Such foundress associations are predicted, by iterated public goods game expansion of LMC theory^48^, to select for strong female bias, due to cooperative sex allocation (whereby all foundresses benefit by the production of only the lowest number of males required for insemination, thus utilizing the maximum host resource for female production). Due to decreasing likelihood of long-term cooperation by all females in larger groups, the type of gradual response to foundress number that we have observed (Fig. 5) is also predicted^48^. While this theory provides a candidate alternative explanation for *S. harmandi* sex ratios, for it to operate foundresses would have to recognize the sex ratio of eggs on the host and to respond by adjusting their own sex allocation decisions (a form of policing against cheating by some foundresses laying higher proportions of male eggs^48^); hymenopteran parasitoids generally do not have this ability^53^ and the egg stage of *S. harmandi* clutches is short relative to the prolonged period of brood tending through the larval stages^16^,^17^. Although the sex of developing larvae may be more readily detectable by tending foundresses^53^, offspring mortality was unrelated to foundress number in *S. harmandi* so it does not seem that infanticide is common (see also Hu *et al*.^16^) (in contrast to solitary and subsocial bethylids^11^,^54^), making post-ovipositional sex ratio adjustment of *S. harmandi* brood sex ratios a less likely explanation for sex ratio bias than LRE, although both selective mechanisms could potentially operate simultaneously.

While *S. harmandi* foundresses care for each other’s offspring, females that have not laid their own eggs do not exhibit brood care^16^,^17^. The conditional occurrence of brood care by adult females suggests that (largely selfish) behaviours, such as reproductive dominance and skew, may operate during the phase of (apparently) cooperative brood care^7^,^52^,^55^,^56^. However, these may be tempered by mutual policing and/or by foundress–foundress relatedness^52^,^55^, especially since in some bethylids adults can recognize kin^57^ probably using variation in cuticular hydrocarbon profiles^58^. Close relatedness between foundresses may be enhanced by codispersal and foraging by females from a given natal patch^34^ and would be expected to select for more greatly female-biased sex ratios^9^,^37^,^53^,^59^,^60^. Cofoundress relatedness, which will be highest when females derive from broods produced by fewer mothers^61^, should also select, via inclusive fitness gains, for altruistic brood care^7^, and this may explain why female *S. harmandi* that have not laid eggs do not commit ovicide^16^,^17^.

In summary, social behaviour in many hymenopterans is highly advanced^3^,^4^,^6^,^7^, but most parasitoid wasps are socially solitary. In parasitic bethylids, both subsociality (brood guarding^11^) and the much less-common quasisociality (cooperative brood care (Fig. 1)) can be explained by direct fitness gains to foundress females: selfish defence of own offspring^11^ and mutually beneficial host suppression, respectively. Female-biased sex ratios in subsocial bethylids can be largely explained by LMC^35^,^36^,^46^ but the ultra-biased sex ratios of the quasisocial *S. harmandi* are better explained by LRE via multifoundress mutually beneficial host exploitation, an unusual scenario among parasitoid wasps. These results do not preclude that the social behaviours of *S. harmandi* may also be mediated by additional effects of kinship between foundresses or that sex allocation may be contingent on some degree of mate competition between males; indeed these influences are expected^9^,^36^,^57^,^58^ and would be especially likely if female offspring disperse from depleted natal hosts in cohesive groups^32^,^60^.

## Methods

### Parasitoids and hosts

**Figure 2:**
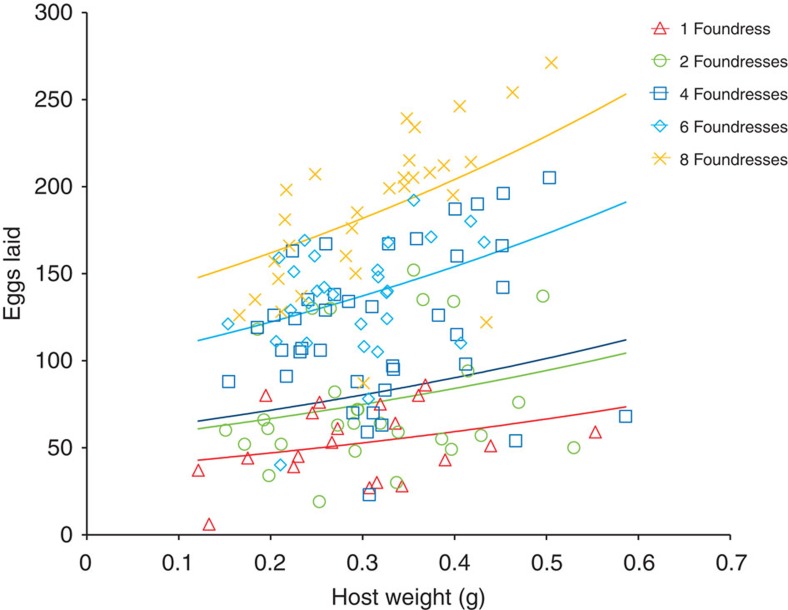
Number of eggs laid on hosts in relation to host size and foundress number. Larger clutches of eggs were laid on heavier hosts and, additively, by larger numbers of foundresses (log-linear ANCOVA: host weight, *F*_1,140_=15.6, *P*<0.001; foundresses, *F*_4,141_=50.64, *P*<0.001; interaction, *F*_4,136_=0.45, *P*=0.773). The fitted regression lines are: number of eggs=exp (1.156 × host weight+constant), with the constants for 1, 2, 4, 6 and 8 foundresses as 3.62, 3.97, 4.04, 4.575 and 4.856, respectively. Sample sizes for 1, 2, 4, 6 and 8 foundresses were 20, 29, 39, 28 and 30, respectively.

**Figure 3:**
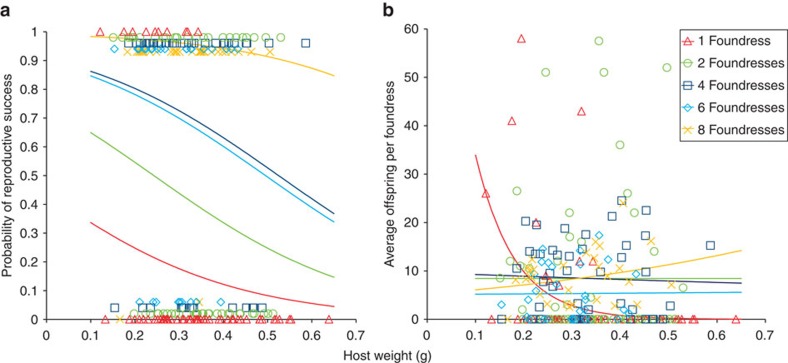
Mutualism in host exploitation. (**a**) The probability of adult offspring being produced declines as host size increases and is additively higher for larger foundress groups (logistic ANCOVA: host weight, *G*_1_=6.38, *P*=0.012, foundresses, *G*_4_=17.06, *P*<0.001, interaction, *G*_1_=1.75, *P*=0.137, *n*=220). The fitted regression lines are: probability of reproductive success=1/(1+(1/(exp (−4.32 × host weight−constant)))) with the constants for 1, 2, 4, 6 and 8 foundresses as 0.246, 1.048, 2.267, 2.139 and 4.52, respectively. (**b**) A larger foundress group size also favours individual foundresses, in terms of mean offspring produced, provided that the host is large; when a host is medium sized, a foundress is likely to produce the most offspring when exploiting it with a smaller group of cofoundresses, or, if the host is small, alone (log-linear ANCOVA: host weight, *G*_1_=3.23, *P*=0.074; foundresses, *G*_4_=3.00, *P*=0.019, interaction: *G*_4_=4.80, *P*<0.001, *n*=220). The fitted regression lines show the mean offspring production per foundress for each number of foundresses: 1 foundress=exp (−12.4 × host weight+4.764), 2 foundresses=exp (host weight+2.132), 4 foundresses=exp (−0.39 × host weight+2.265), 6 foundresses=exp (0.13 × host weight+1.634), 8 foundresses=exp (1.54 × host weight+1.654). In both panels, sample sizes for 1, 2, 4, 6 and 8 foundresses were 60, 55, 45, 30 and 30, respectively.

**Figure 4:**
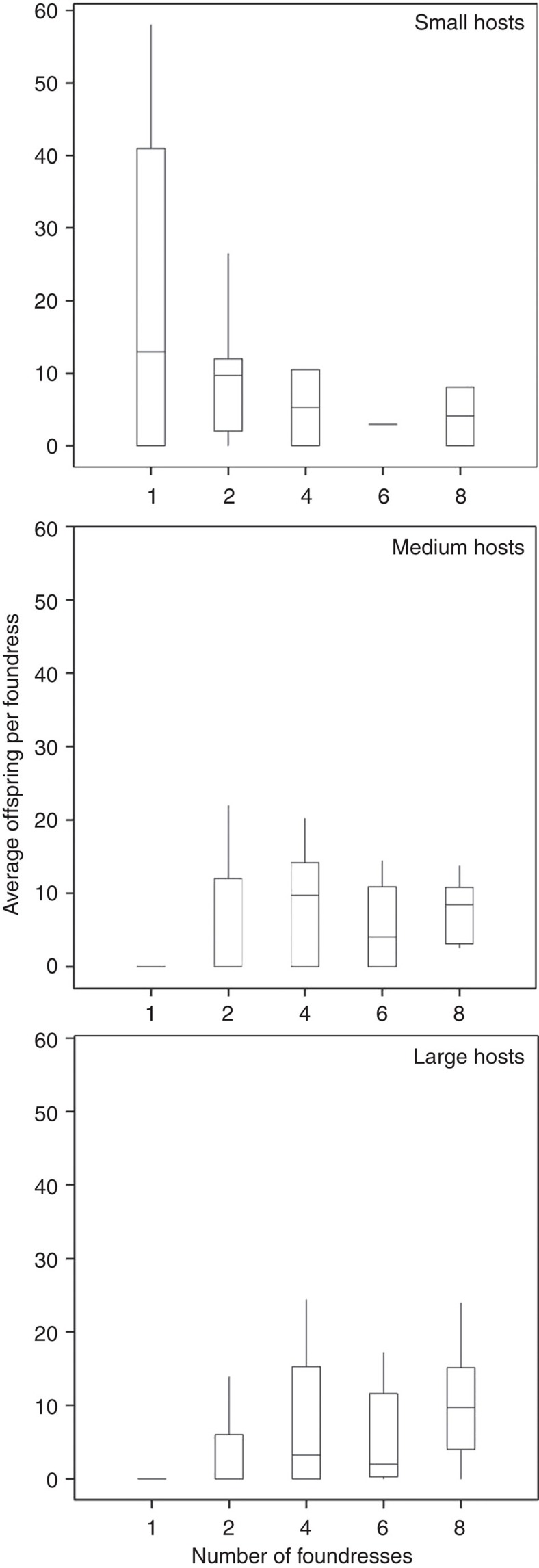
Box-and-whisker plots of offspring production according to foundress number within each host-size category. Hosts were classified *post hoc* as small (≤ *ca.* 0.2 g, *n*=17), medium (*ca.* 0.2–0.33 g, *n*=105) and large (>0.33 g, *n*=98). Boxes show median (centre) and interquartile ranges (ends) for offspring production for each number of foundresses, with whiskers indicating the variability within each category. Differences in per foundress offspring production within each host-size category were not significant for small hosts, due to limited sample size (Kruskal–Wallis (KW) test: *H*=1.229) or for medium hosts (KW: *H*=2.676, *P*=0.613). For large hosts, production differed significantly (KW: *H*=31.47, *P*<0.001) due to groups of eight foundresses being more productive than one-foundress and two-foundress groups and also to four-foundress groups being more productive than single foundresses (*P*<0.05 in all three *post hoc* multiple comparison tests). Overall, the size of the foundress group that maximizes the median *per capita* production of offspring increases as host size increases.

The life histories of parasitoid wasps have been instrumental in stimulating evolutionary and ecological theory, such as sex ratio evolution^9^,^10^,^12^,^33^,^35^,^62^, but most are socially solitary^10^, lacking parental care. *Sclerodermus* species (members of the hymenopteran family Bethylidae) are unusual among parasitoids in that multiple females may attack and oviposit on a single host and then remain together tending the developing brood^13^,^14^,^16^,^17^,^18^,^21^,^25^,^30^,^31^,^32^. In common with its congeners, *S. harmandi* (Buysson), which has been considered synonymous with *S. guani*^63^, lays clutches of eggs onto the hosts integument, typically after first feeding on the host^26^,^27^ with clutch sizes ranging up to around 100 eggs, dependant on the size, developmental stage and species of the attacked host^26^,^28^,^44^ (Fig. 2). The lifetime fecundity of individual females ranges from 30 to 200 eggs^64^.

**Figure 5:**
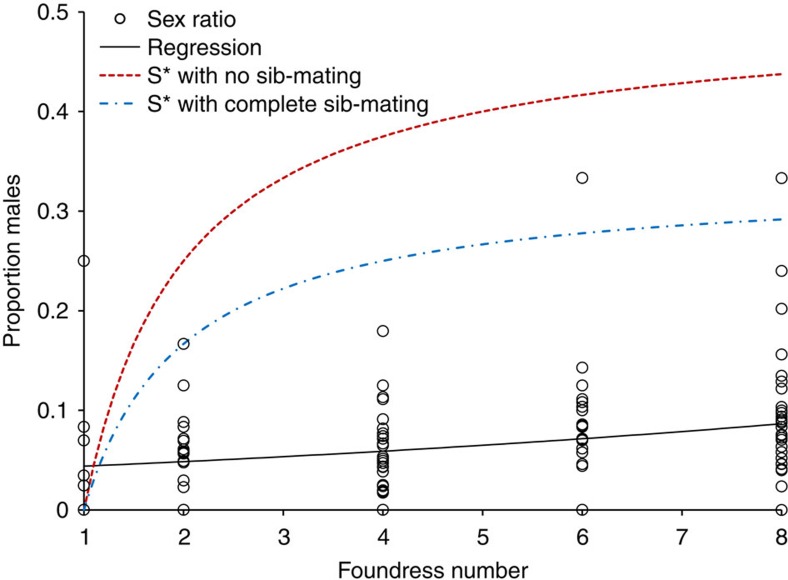
Extreme sex ratio bias in *Sclerodermus harmandi.* Sex ratios are more biased than predicted by classical LMC theory. The lower line is the fitted logistic regression of sex ratio on foundress number (*F*_1,108_=15.79, *P*<0.001, *n*=110, proportion of offspring that are male=1/(1+(1/(exp (0.1033 × host weight −3.184)))) and the upper lines show the evolutionarily stable sex ratio (S*) response to foundress number (*N*) in haplodiploid species in single-generation mating groups with either no sib-mating (top dashed line: S*=[*N*−1]/2*N*) or complete sib-mating (middle dotted and dashed line: S*=[*N*−1]/3*N*)^9^,^35^.

Progeny sex ratios are biased towards females^28^,^32^ (Fig. 5). Males typically mate with maturing broodmate females when these emerge or prior to their emergence by chewing entrances into their cocoons^16^,^64^. Adult wing dimorphisms (alate and apterous forms) occur in both sexes in the genus^13^,^45^,^65^. In *S. harmandi* apterous males are rare^32^ but possession of wings does not necessarily indicate a propensity or an ability to fly^13^,^18^. Females are larger in body size than males (female: 3.5 mm in body length, apterous and 3.2 mm, alate; male: 2.1 mm, alate)^65^. Males live for around 1 week but females typically live for 2–7 months^32^. Mated females overwinter in groups in host-made tunnels or cavities in trees^21^,^32^.

*S. harmandi*, a widely used biological control agent in forest pest-control practices in mainland China^66^, mainly parasitizes multiple wood-boring long-horned beetles (Cerambycidae) in nature^65^, which varies widely in larval body size depending on host species (for example, larval *Monochamus alternatus* from 200 to 700 mg in body weight were parasitized in the field^28^). These hosts often have life histories of 1 year or more (uni-voltinism and semi-voltinism), but *S. harmandi* is multivoltine^64^. Natural rates of host availability and parasitism are poorly known due to difficulties in sampling within woody tissues; available estimates range from 0.015 (ref. 20) to 4% (ref. 32). Following inundative releases of *S. harmandi* in biological pest-control programmes, parasitism of hosts in the field may reach 50–80% (refs 21, 64, 66).

For this study, *S. harmandi* was cultured at the Forestry Institute of Jiangsu Province, PR China, where it is mass produced for the biocontrol of the pine sawyer beetle *Monochamus alternatus* Hope (Coleoptera: Cerambycidae), a pest of conifers which vectors nematodes causing pine wilt disease^21^,^26^,^27^,^64^,^66^. *M. alternatus* hosts were collected from pine forests in Liyang County during the winter of 2011, and maintained in the refrigerator (*ca.* 10 °C).

### Experiment

Laboratory experiments were conducted at 25 °C and 60–80% r.h. in 2012 at Nanjing Agricultural University. Varying numbers of adult female *S. harmandi* (foundresses) were presented with a larval *M. alternatus* host, weighed to an accuracy of 0.0001, g (Mitler, AL204-IC) in a glass vial (1.0 cm diameter, 5.0 cm long): foundress numbers were 1, 2, 4, 6 or 8 (*n*=60, 55, 45, 30 and 30, respectively, giving 220 replicates and adequate overall statistical power). Hosts and parasitoids were examined twice daily and the occurrence of oviposition, the time to oviposition, the number of eggs laid, the duration of offspring development and number of male and female offspring produced were recorded. The average weight of females within offspring groups was also recorded to an accuracy of 0.0001, g.

### Statistical analysis

Generalized linear modelling (in Genstat, V14.1, VSN International) was used to explore the influences of host size and foundress number on offspring production and sex allocation, using backwards elimination of explanatory variables from initial statistical models and with overdispersion taken into account, via empirical estimation of scaling parameters, where appropriate^67^,^68^. Subanalyses within host–weight categories used Kruskal–Wallis tests with *post hoc* multiple comparison testing^69^.

## Author contributions

B.L. designed the experiment, X.T. and L.M. performed the experiment, F.X. supplied biological material, A.K. and I.C.W.H. analysed the data and wrote the manuscript. All authors discussed and approved the manuscript.

## Additional information

**How to cite this article**: Tang, X. *et al*. Mutually beneficial host exploitation and ultra-biased sex ratios in quasisocial parasitoids. *Nat. Commun.* 5:4942 doi: 10.1038/ncomms5942 (2014).
